# Response to trametinib treatment in progressive pediatric low-grade glioma patients

**DOI:** 10.1007/s11060-020-03640-3

**Published:** 2020-10-07

**Authors:** Florian Selt, Cornelis M. van Tilburg, Brigitte Bison, Philipp Sievers, Inga Harting, Jonas Ecker, Kristian W. Pajtler, Felix Sahm, Annabelle Bahr, Michèle Simon, David T. W. Jones, Lennart Well, Victor-Felix Mautner, David Capper, Pablo Hernáiz Driever, Astrid Gnekow, Stefan M. Pfister, Olaf Witt, Till Milde

**Affiliations:** 1 Hopp Children’s Cancer Center (KiTZ), Im Neuenheimer Feld 430, 69120 Heidelberg, Germany; 2 Clinical Cooperation Unit Pediatric Oncology, German Cancer Research Center (DKFZ) and German Consortium for Translational Cancer Research (DKTK), Heidelberg, Germany; 3 KiTZ Clinical Trial Unit (ZIPO), Department of Pediatric Hematology and Oncology, Heidelberg University Hospital, Heidelberg, Germany; 4 Institute of Diagnostic and Interventional Neuroradiology, University Hospital Wuerzburg, Würzburg, Germany; 5 Department of Neuropathology, Institute of Pathology, Heidelberg University Hospital, Heidelberg, Germany; 6 Clinical Cooperation Unit Neuropathology, German Consortium for Translational Cancer Research (DKTK), German Cancer Research Center (DKFZ), Heidelberg, Germany; 7 Department of Neuroradiology, Heidelberg University Hospital, Heidelberg, Germany; 8 Department of Pediatric Oncology/Hematology, Charité Universitaetsmedizin Berlin, Corporate Member of Freie Universitaet Berlin, Humboldt-Universitaet zu Berlin and Berlin Institute of Health, Berlin, Germany; 9 Department of Diagnostic and Interventional Radiology and Nuclear Medicine, University Medical Center Hamburg-Eppendorf, Hamburg, Germany; 10 Department of Neurology, University Medical Center Hamburg-Eppendorf, Hamburg, Germany; 11 Department of Neuropathology, Corporate Member of Freie Universitaet Berlin, Humboldt-Universitaet Zu Berlin, and Berlin Institute of Health, Charité-Universitaetsmedizin Berlin, Berlin, Germany; 12 German Cancer Consortium (DKTK), German Cancer Research Center (DKFZ), Partner Site Berlin, Berlin, Germany; 13 Swabian Children’s Cancer Center, University Hospital Augsburg, Augsburg, Germany; 14 Division of Pediatric Neurooncology, German Cancer Research Center (DKFZ) and German Consortium for Translational Cancer Research (DKTK), Heidelberg, Germany; 15 Pediatric Glioma Research Group, German Cancer Research Center (DKFZ), Heidelberg, Germany

**Keywords:** Pediatric low-grade glioma, Targeted therapy, BRAF, NF1, MAPK pathway, MEK inhibitor, Trametinib

## Abstract

**Introduction:**

A hallmark of pediatric low-grade glioma (pLGG) is aberrant signaling of the mitogen activated protein kinase (MAPK) pathway. Hence, inhibition of MAPK signaling using small molecule inhibitors such as MEK inhibitors (MEKi) may be a promising strategy.

**Methods:**

In this multi-center retrospective centrally reviewed study, we analyzed 18 patients treated with the MEKi trametinib for progressive pLGG as an individual treatment decision between 2015 and 2019. We have investigated radiological response as per central radiology review, molecular classification and investigator observed toxicity.

**Results:**

We observed 6 partial responses (PR), 2 minor responses (MR), and 10 stable diseases (SD) as best overall responses. Disease control rate (DCR) was 100% under therapy. Responses were observed in KIAA1549:BRAF- as well as neurofibromatosis type 1 (NF1)-driven tumors. Median treatment time was 12.5 months (range: 2 to 27 months). Progressive disease was observed in three patients after cessation of trametinib treatment within a median time of 3 (2–4) months. Therapy related adverse events occurred in 16/18 patients (89%). Eight of 18 patients (44%) experienced severe adverse events (CTCAE III and/or IV; most commonly skin rash and paronychia) requiring dose reduction in 6/18 patients (33%), and discontinuation of treatment in 2/18 patients (11%).

**Conclusions:**

Trametinib was an active and feasible treatment for progressive pLGG leading to disease control in all patients. However, treatment related toxicity interfered with treatment in individual patients, and disease control after MEKi withdrawal was not sustained in a fraction of patients. Our data support in-class efficacy of MEKi in pLGGs and necessity for upfront randomized testing of trametinib against current standard chemotherapy regimens.

## Introduction

Low-grade gliomas (pLGG) are the most common brain tumors in children and account for about 30% of all pediatric brain tumors [1, 2]. Standard of care (SOC) treatment options such as surgery, followed by chemotherapy and occasionally radiotherapy (RT) where indicated, have been shown to be effective, leading to 10-year overall survival (OS) and event-free survival (EFS) of 94% and 44%, respectively [3]. Chemotherapy is currently recommended as first-line, nonsurgical treatment for most patients [3, 4]. Although the disease control rate is excellent (> 90%) for all chemotherapy regimens, up to 80% of incompletely resected tumors progress and require one or more lines of adjuvant therapy. Many patients suffer from extensive tumor and treatment related morbidity, e.g. visual function loss, motor deficits, deafness, developmental delay, vasculopathy, and hypopituitarism [5]. Endocrinopathies, developmental abnormalities, and neurocognitive dysfunction are common late effects after RT [6, 7], and are more pronounced in younger children and patients with neurofibromatosis type 1 (NF1), who are specifically at risk for RT-induced vascular malformations [8] and secondary malignancies [9]. It is currently recommended to avoid or at least defer RT by repeated lines of chemotherapy [10], especially in younger patients and patients with NF1. New tailored therapy approaches are needed to improve the long-term outcome of pLGG-patients and reduce therapy- and disease-related morbidity.

The majority of pLGG show oncogenic activation of the mitogen activated protein kinase (MAPK) signaling pathway [11–13]. Pilocytic astrocytoma (PA) is the most frequent subgroup of pLGG, accounting for about half of all pLGG cases [2], with nearly 100% of PAs harboring an activating alteration of the MAPK pathway [14]. The most frequent aberration is the *KIAA1549:BRAF*-fusion, beside less frequent alterations such as other *BRAF* fusions*, BRAF* mutations*, FGFR1* mutations*, NF1* mutations*, KRAS* mutations and *PTPN11* mutations or *NTRK2* fusions [12, 14]. The universal activation of the MAPK pathway makes pLGGs uniquely suited for targeted treatment approaches. Pre-clinical data points to robust inhibition of the MAPK pathway by MEK inhibitors (MEKi) such as selumetinib and trametinib in pLGG cells with *KIAA1549:BRAF*-fusion or *BRAF V600E* mutation [15, 16]. Selumetinib has been studied in a series of clinical trials: 20% of patients with recurrent or progressive pLGG showed sustained partial response in a phase I clinical trial [17] and 36–40% of progressive pLGG patients experienced a sustained partial response in a phase II trial [18]. Selumetinib also showed significant activity in NF1-associated plexiform neurofibromas (PNF), which are otherwise resistant to systemic therapy, leading to the first approval of a MEKi in a specific pediatric indication [19].

Trametinib is currently being tested in phase I/II clinical trials (NCT02124772) and first limited data is available in abstract format for patients with BRAF fusions [20]. The published data on the activity of trametinib in pLGG is derived from case series [21–24]. Two case series reported on two and six progressive PA patients with *KIAA1549:BRAF*-fusion [21, 23], with two partial responses (PR) in one study [23], and two partial responses (PR), three minor responses (MR) and one progressive disease (PD) in the second [21]. The third case series [24] reports on 14 low- and high-grade pediatric MAPK-altered brain tumors of different histologies (11 low-grade and 3 high-grade tumors) treated with trametinib alone or in combination. Eight of the 11 patients with low-grade tumors received trametinib monotherapy, of which four had a *BRAF*-fusion, one had a *BRAF V600E* mutation, one an *NF1* alteration and two no detected MAPK alteration. In these seven tumors treated with trametinib monotherapy, best responses were three PRs, two PD, one SD, and two missing responses due to early treatment stop because of toxicity. The most recently published case series [22] reported on eleven pLGG patients treated with trametinib, four of which had a *KIAA1549:BRAF*-fusion, four an *NF1* mutation, one had an *FGFR* mutation and one had a *CDKN2A* loss. The underlying molecular alteration was unknown in one patient. The authors reported on two PRs, two MRs and six SDs under trametinib treatment.

Several clinical trials will evaluate trametinib prospectively in pLGGs. The TRAM-01 trial [25] is a phase II multicentric open-label basket trial including four groups (NF1 LGG, NF1 PNF, BRAF-fusion LGG, other MAPK-acitvated glioma). The upcoming LOGGIC Europe trial is the first prospective randomized clinical trial to compare trametinib to the SOC carboplatin/vincristine and to vinblastine monotherapy in newly diagnosed pediatric LGG patients.

Here we report the results of a retrospective centrally reviewed multi-center study of the activity and toxicity of trametinib in the largest pLGG series to date, consisting of 18 patients with molecularly characterized pLGGs, eight with NF1-related and ten with sporadic BRAF-fusion- positive, BRAF V600E-positive or FGFR-mutated pLGGs.

## Methods

A retrospective multi-center analysis of pLGG patients treated with trametinib in “off-label-use” in eight centers in Germany between 2015 and 2019 was performed. Consent for data collection was obtained by inclusion of patients in clinical and diagnostic studies, either the SIOP-LGG-2004 trial (NCT00276640), the LGG registry or PTT2.0 (NCT-2016-0414; DRKS00011707). Participation in SIOP-LGG-2004 trial and registry allowed for retrospective tissue and MRI analyses beyond the completion of trial-required components. Molecular work-up of the tumors in cases with available material had either already been done by the treating centers or via PTT2.0 or was performed retrospectively on archived tissue material. Molecular classification was done by DNA methylation array [26]. *NF1* alterations were detected by sequencing of the *NF1* locus or gene panel sequencing [27]. *BRAF* and *FGFR1* mutations were detected by gene panel sequencing [27]. *BRAF*-fusions were detected by FISH, gene panel sequencing, copy number plot analysis, or targeted PCR. Grading of therapy related adverse events (AE) was retrospectively done according to Common Terminology Criteria for Adverse Events (CTCAE) V5.0 [28]. Response assessment by MRI to trametinib treatment was retrospectively centrally reviewed at the Neuroradiological Reference Center for HIT-Studies in Würzburg, Germany, and evaluated in accordance with the SIOP-LGG study guidelines described in the SIOPE-BTG and GPOH Guidelines for Diagnosis and Treatment of Children and Adolescents with Low Grade Glioma [4] and the more recently published RAPNO criteria for LGG [29]. The tumor volume was calculated using the (ellipsoid volume) formula ½ (AxBxC), where A, B and C are the maximum dimensions in the standard planes.

All sequences available were used for evaluation. For measurement the sequence best depicting the tumor extent was used, i.e. primarily T2 or T2 FLAIR in two planes in case of partly or non-enhancing tumors and T1 images post contrast only for completely enhancing tumors. Due to the retrospective nature of the study, not all MRIs were conducted according to the same standards including standard sequences and forced deviations from RAPNO recommendations in cases where tumors had to be measured using T1 images post contrast images. Responses were calculated in relation to pre-treatment baseline MRIs. The response criteria were defined as follows: complete response (CR) defined as no evidence of residual or recurrent tumor or dissemination; partial response (PR) defined as reduction of tumor volume ≥ 50% and no new lesions; minor response (MR) defined as reduction in tumor volume between 25 and 50% without new lesions; stable disease (SD) defined as change in tumor volume between +25% and − 25% without new lesions, and progressive disease (PD) defined as ≥ 25% increase in tumor size or appearance of new lesions. Tumors that were not able to be captured by the ellipsoid, as it was the case for most opticohypothalamic tumors, were rated as non measureable but eligible according to the LGG Guideline [4] and RAPNO LGG [29]. The volume had to be estimated to the best knowledge and experience of the reference center. This estimation has been correlated with calculation of measureable representative parts of the tumor. Disease control rate (DCR) was defined as the sum of CR, PR, MR and SD. Response of NF1-related non-pLGG lesions (neurofibromas (NFs) or PNFs), if present, to trametinib treatment was documented but not centrally reviewed. Volume of PNFs in one NF1 patient (patient 6) was determined by MedX software (v3.42). MedX utilizes a heuristic-based semi-automated method for segmentation and measurement and assessment has been proven as sensitive and reproducible, yielding results similar to manual tracings of tumor margins [30].

## Results

### Patient characteristics and prior treatments

**Fig. 1:**
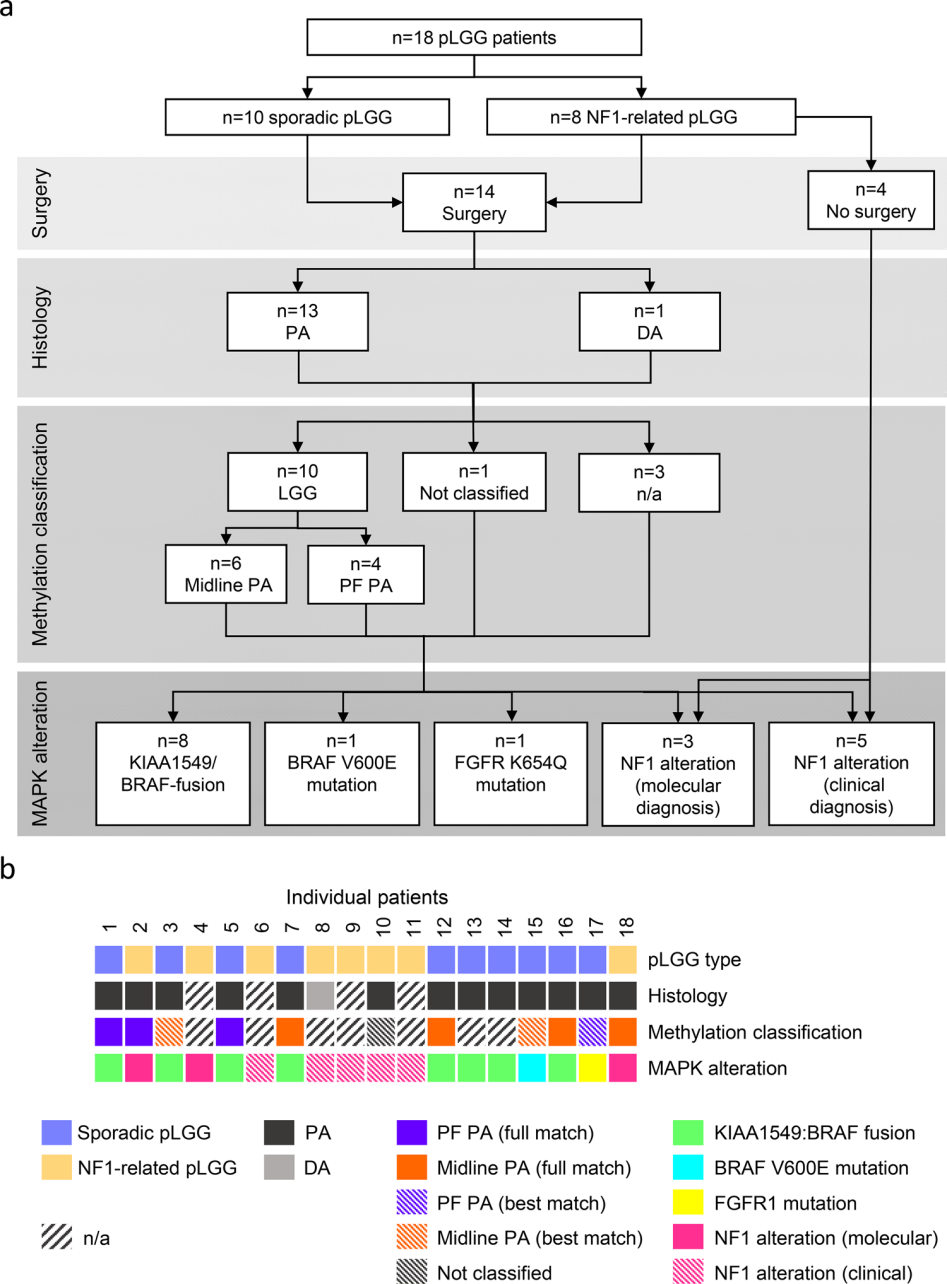
Overview of diagnostic and molecular work-up **a** Diagnostic and molecular work-flow **b** Graphical summary of diagnostic and molecular features for each patient included. *PA* pilocytic astrocytoma, *DA* diffuse astrocytoma, *PF PA* subclass posterior fossa pilocytic astrocytoma, *Midline PA* subclass midline pilocytic astrocytoma; “full match”, successful methylation classification; “best match”, methylation classification score below cut-off but highest similarity to the indicated methylation class/subclass; Not classified, no match with any of the methylation reference classes; *n/a* not applicable, testing not performed because no tumor material was available. Of note, molecular NF1 testing was performed in blood and tumor in two patients (patient 2 and 18) and only in blood in one patient (patient 4)

Patients’ characteristics are shown in detail in Table 1. Median age at diagnosis was 2.1 years (range: 0.5–9.9). The diagnostic and molecular work-up is shown in Fig. 1a and diagnostic and molecular data is summarized in Fig. 1b. Ten of 18 (56%) patients had sporadic pLGG and 8/18 (44%) patients had NF1-related pLGG. Histology was available for 14/18 (78%) patients while 4/18 (22%) patients did not undergo surgery and diagnosis was made based on clinical criteria of NF1 and typical radiology features by central reference review. Among the patients with available histology, 13 were diagnosed with PA and one was diagnosed with diffuse astrocytoma (DA). DNA methylation data was already available for 8/18 (44%) patients. DNA methylation arrays were retrospectively added in 3/18 (17%) patients (patients 10, 15 and 17) leading to 11/18 (61%) patients with DNA-methylation data. In total 7/18 (38%) patients were classified as low-grade gliomas by methylation array (full match). 3/18 (17%) showed the highest similarity to LGG according to DNA-methylation or tSNE clustering although the methylation scores were below the cut-off (best match). One tumor could not be classified by DNA methylation array (patient 10). Information on the underlying MAPK alteration was already available for 11/18 (61%) patients and was retrospectively detected in 2/18 (11%) patients (patients 15 and 17) leading to a total of 13/18 (72%) of patients with molecularly detected MAPK alteration. Alterations detected included 8 *KIAA1549*:*BRAF*-fusions, three *NF1* alterations, one *BRAF V600E* mutation and one *FGFR1 K654Q* mutation. Of note, molecular *NF1* testing was performed in blood and tumor in two patients (patient 2: p.514_514del, and patient 18: p.888fs, both detected by gene panel sequencing) and only in blood in one patient (patient 4: p.1153fs detected by targeted sequencing of the NF1 gene including exon-flanking intronic regions). The remaining 5/18 (33%) patients, who either did not undergo surgery or did not have remaining material, all had a clinical diagnosis of NF1 and an *NF1* alteration could be assumed.

**Table 1:** Patients’ characteristics

Number of patients included, n (%):	18 (100)
Median age at diagnosis, years (range):	2.1 (0.5–9.9)
Sex, n (%)	
Male	8 (44)
Female	10 (56)
Neurofibromatosis type 1, n (%)	
Yes	8 (44)
No	10 (56)
Tumor localization, n (%)	
OPG	12 (66)
OPG + brain stem	1 (6)
Brain stem	4 (22)
Cranio-cervical	1 (6)
Any prior pLGG-related treatment	
Yes	16 (89)
No	2 (11)
Surgery, n (%)	
> 1 partial resection	3 (17)
1 partial resection	7 (38)
Biopsy	3 (17)
Cyst fenestration	1 (6)
No surgery	4 (22)
Number of prior chemotherapy lines, n (%):	
> 3	7 (38)
3	4 (22)
2	1 (6)
1	4 (22)
0	2 (11)
Prior radiotherapy, n (%)	
No	16 (88)
Yes	1 (6)
Radiosurgery	1 (6)

*PA* pilocytic astrocytoma, *DA* diffuse astrocytoma, *OPG* optico-hypothalamic glioma

Sixteen of 18 (89%) patients had received prior treatment for pLGG including surgery, chemotherapy, radiotherapy, or combinations thereof. Ten of 18 (55%) patients had at least one tumor volume reductive surgery. Sixteen of 18 (89%) patients had at least one prior line of chemotherapy and 7/18 (38%) had more than three chemotherapy lines (range: four to ten). Regimens used included carboplatin/vincristine (according to the SIOP-LGG 2004 protocol), vinblastine monotherapy, and less common protocols. One patient had received proton beam radiotherapy and one gamma knife radiosurgery. Two NF1 patients had no prior pLGG-related treatment.

### Targeted treatment with trametinib and response to treatment

**Fig. 2:**
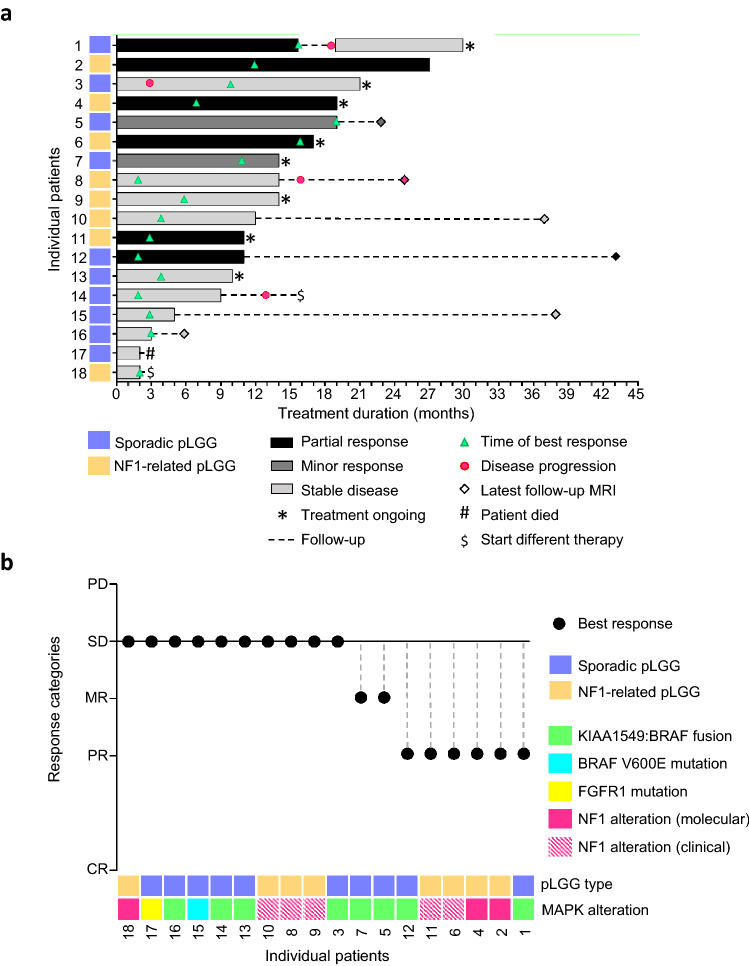
Response to trametinib treatment **a** Swimmer plot demonstrating the duration of exposure to trametinib analyzed by centrally reviewed best overall response. **b** Best responses depicted in categories (*PD* progressive disease >  + 25% size change, *SD* stable disease, between + 25% and − 25% size change; *MR* minor response, between > − 25 and < − 50% size change, *PR* partial response, > 50% size change, *CR* complete response, − 100% size change) for each individual patient in the context of pLGG type and underlying molecular alteration. Of note, molecular NF1 testing was performed in blood and tumor in two patients (patient 2 and 18) and only in blood in one patient (patient 4)

Eighteen pLGG patients received trametinib for their progressive tumor. All 18 patients presented with an indication to therapy due to MRI-morphological tumor progression when trametinib therapy was initiated and either had developed new neurological symptoms or were at risk of developing tumor related symptoms due to the location of their tumor. Targeted therapy was initiated after failure of prior SOC treatments in 16/18 (89%) patients, while two patients with NF1-associated pLGG 2/18 (11%) and clinical significant PNFs had not received prior SOC. Details on trametinib treatment and treatment response are summarized in Table 2. All tumors were radiologically evaluable but most tumors were not measurable due to irregular shape precluding exact volumetry. Response was categorized in response categories (SD, MR, PR, CR). Best overall responses were observed after a median treatment time of 4 (range: 1–19) months and included 6 PR, 2 MR and 10 SD (Fig. 2a, b). MR and PR was observed in KIAA1549:BRAF-fusion- and NF1-driven pLGG (Fig. 2b). Disease control rate (DCR) during treatment with trametinib was 100%. One PD was observed retrospectively by central radiological review after 3 months of trametinib treatment (patient 3). The increase in volume was slightly above + 25% according to central review and had not been assessed as PD by local radiology at the time of treatment. Therefore, treatment was continued and the patient then showed SD compared to baseline tumor volume assessment in the further course. The median duration of treatment was longer in the group of patients with either MR or PR (median 16.5; range 11–27 months) as compared to patients with SD as best overall response (9.5; 2–21 months). Patients with MR or PR had lower numbers of prior chemotherapy lines (median: 1.5; range: 0–5 months) compared to patients with SD (5; 1–10). No differences in response based on age at trametinib onset, localization of the tumor, histology or underlying molecular alteration were observed.

**Table 2:** Treatment with trametinib, response, and follow-up after end of treatment

Median age at trametinib onset, years (range)	8.2 (3.5–17.3)
Status before treatment, n (%)	
Progressing tumor	14 (78)
Progressing tumor with visual impairment	2 (11)
Progressing tumor with worsened neurological symptoms	2 (11)
Mean trametinib dose, mg/kg*day (SEM)	0.03 (± 0.009)
Median treatment time, months (range)	12.5 (2–27)
Best overall response, n (%)	
PR	6 (33)
MR	2 (11)
SD	10 (56)
PD	0 (0)
Disease control rate, n (%)	18 (100)
Time to best overall response, months (range)	4 (1–19)
Treatment status, n (%)	
Ongoing	7 (38)
Stopped	10 (56)
Re-initiated and ongoing after end of treatment	1 (6)
Reason for discontinuation of trametinib, n (%)	
Sustained response/SD	2 (11)
Planned EOT/decision of treating physician	2 (11)
Treatment related side effects	2 (11)
Further loss of vision	1 (6)
Increasing tumor volume	2 (11)
N/A	1 (6)
Last status after EOT (10 patients; median follow up 7 (1–33) months), n (%)	
PR, no further treatment	1 (6)
MR, no further treatment	1 (6)
SD, no further treatment	3 (17)
PD, no further treatment	1 (6)
Progression, different treatment initiated	1 (6)
Increase in tumor size, different treatment initiated	1 (6)
Death	1 (6)
N/A	1 (6)

*SEM* standard error of mean, *PR* partial response, *MR* minor response, *SD* stable disease, *PD* progressive disease, *EOT* end of treatment, *N/A* not applicable

### Follow-up after end of treatment

Ten of 18 (56%) patients had stopped trametinib therapy at the end of data collection, and 8/18 (44%) were still (n = 7) or again (n = 1; re-initiation due to progression after EOT) on treatment (Table 2; Fig. 2a). One patient stopped trametinib just before the end of data collection and no follow-up (FU) data was available (patient 2). The median FU time of the 9/10 patients after EOT was seven (range 1–33) months. One patient (patient 17, clinical PD) died of the disease 1 month after EOT, accompanied by multiple severe complications related to either the disease (hyponatremia) or prior therapies (oto-, hemato-, and cardiac toxicities; infection of intraventricular reservoir causing severe meningoencephalitis). One patient (patient 14) received vinblastine 7 months after EOT due to disease progression and another patient (patient 18) received bevacizumab 1 month after EOT due to increase in tumor size (not PD). Six patients had no further treatment. One sustained PR (31 months after EOT), one sustained MR (4 months after EOT), three sustained SD (three, 25 and 33 months after EOT, respectively) and one PD (patient 8, 2 months after EOT, without need for treatment due to spontaneous stabilization 10 months after EOT) were observed during FU. NF1-related non-pLGG tumors (NFs/PNFs) of three NF1 patients showed no change in tumor volume during trametinib treatment (SD; not centrally reviewed) and volumetry of PNFs in another NF1 patient (patient 6) showed a reduction of tumor volume by 26% under treatment (not shown).

### Progression after trametinib withdrawal

Three of 11 (27%) patients (patients 1, 8 and 14; Fig. 2a) who stopped trametinib treatment showed progression after the end of treatment within two to four months. One tumor (patient 1; Fig. 2a) had initially shown PR during the first round of treatment, and stabilization (SD) was observed after re-initiation of trametinib treatment upon PD.

### Trametinib-related adverse events

Sixteen of 18 (89%) experienced at least one trametinib-related AE. The most frequent AEs were skin-related, with maculopapular rash, paronychia, acneiform rash and xerodermia being the most commonly reported AEs (Table 3). At least one CTCAE v5.0 grade III or IV AE was reported in 8/18 (44%) patients. This included acneiform rash (17%), erysipelas (11%), maculopapular rash (11%), paronychia (6%), eczema (6%), dermatitis bullosa (6%) and pancreatitis (6%). Of note, one patient experienced a pneumococcal meningitis. However, the relatedness to trametinib treatment remained uncertain since this has not been reported as trametinib-associated AE in larger adult trametinib studies. Most AEs were well manageable by supportive care and/or short treatment interruptions. Dose limiting toxicities occurred in 6/18 (33%) patients and prompted dose reduction to 33–75% of the starting dose. Based on our limited data there was no correlation between dose reduction and treatment response. The severity of the AEs resulted in discontinuation of treatment in 2/18 (11%) patients (acneiform rash in both cases). No treatment related death was observed. AEs were gathered from medical records and may have been underestimated when compared to a prospective clinical trial.

**Table 3:** Treatment related adverse events

	All CTCAE grades	CTCAE grade III/IV
Patients with adverse events, n (%)	16 (89)	8 (44)
Treatment related adverse events, n (%)		
Rash maculopapular	7 (38)	2 (11)
Paronychia	7 (38)	1 (6)
Rash acneiform	5 (28)	3 (17)
Xerodermia	4 (22)	0 (0)
Diarrhea	2 (11)	0 (0)
Dizziness	2 (11)	0 (0)
Eczema	2 (11)	1 (6)
Erysipelas	2 (11)	2 (11)
Fatigue	2 (11)	0 (0)
Oral mucositis	2 (11)	0 (0)
Pruritus	2 (11)	0 (0)
Abdominal pain	1 (6)	0 (0)
Bilirubin elevation	1 (6)	0 (0)
Constipation	1 (6)	0 (0)
Dermatitis bullosa	1 (6)	1 (6)
Infection without causative agent	1 (6)	0 (0)
Left ventricular dysfunction	1 (6)	0 (0)
Pancreatitis	1 (6)	1 (6)
Pneumococcal meningitis	1 (6)	1 (6)
Sinus bradycardia	1 (6)	0 (0)
Dose limiting toxicity, n (%)	6 (33)	
Dose reduction, n (%)	6 (33)	
Adverse event related discontinuation of treatment, n (%)	2 (11)	

*CTCAE* common terminology criteria for adverse events

## Discussion

While safety and efficacy of the MEKi selumetinib in the treatment of pLGG has been well documented [17, 18], there is still only limited data on the MEKi trametinib in the context of pLGG treatment. Selumetinib was shown to be active in different genetic backgrounds including *KIAA1549:BRAF* fusions, *BRAF V600E* mutation and *NF1* mutations in clinical phase 1 and 2 trials [17, 18]. However, activity of trametinib in pLGG has only been described in small retrospective case series and case reports [21–24, 31]. We here report on 18 patients with KIAA1549:BRAF-, BRAF V600E-, FGFR1- or NF1-driven progressive pLGG treated with the MEKi trametinib between 2015 and 2019. Of note, our cohort was mainly comprised of patients with optico-hypothalamic tumors and the median age at diagnosis was low (2.1 years) compared to the median age at diagnosis for all pLGG (7.6 years), thus representing a clinically rather high risk LGG cohort [32]. Two NF1 patients (patients 6 and 11) with additional clinical significant, unresectable plexiform neurofibroma (PNF) received first line trametinib treatment outside of a clinical trial (not available). A PR of the pLGG as well as size reduction of their PNF was seen for both patient 6 (PNF size change: -26%) and 11 (no exact PNF volumetry). MEKi are indeed the only proven effective treatment for PNFs in NF1 patients [33], and have in particular been shown to be efficacious in NF1-related optic pathway gliomas in a phase 2 study [18]. The response rates of NF1-related pLGG treated with selumetinib [18] appear to be similar to the response rates of NF1-related pLGG treated with chemotherapy [34]. Non-approved drugs should not be used outside clinical trials, but first line MEKi treatment could be justified in this particular patient cohort and clearly underscores the urgent need for recruiting clinical trials. Disease control rate was 18/18 (100%) under treatment, and 6/18 (33%) patients showed PR as best overall response. PR was observed in both, KIAA1549:BRAF- and NF1-driven tumors. The responses observed here were comparable to published data on pLGG patients treated with selumetinib where PR was documented as best response in 36%-40% of relapsed pLGG patients [18]. Of note, our study differed in terms of response-assessment in some aspects. This was due to a high rate of evaluable but non-measurable tumors precluding exact volumetry as well as to the retrospective nature of our study where not all MRIs were conducted according to the same standards in regard to both imaging sequences and timepoints. These factors have to be taken into account when comparing our data to response data from other studies. Unlike for selumetinib (reported median time to partial response: 7.54 months [18]) no phase II data on the time to best response after initiation of trametinib treatment in pLGG patients is available. The median time to best response in our cohort was shorter (median: 4, range: 1–19 months) compared to the report of Manoharan et al. (9.8; 3.8–22 months) [22]. However, the median time of treatment was shorter (12.5; 2–27 months) in our cohort compared to the study of Manoharan et.al. (19.2; 3.8–29.8 months). Since best responses to trametinib can occur after as much as 22 months after initiation of treatment [22], the comparably low median total treatment duration of our cohort may underestimate response rates as well as the time to best response. Moreover, in our cohort duration of treatment was longer in the group of patients who experienced at least MR (16.5 (range 11–27) months) as compared to patients with SD as best overall response (median 9.5 (range: 2–21) months), indicating that treatment duration could be a parameter influencing response rates. No patient in our cohort showed CR during/after trametinib treatment. This observation is compatible with most other reports on MEKi showing that CR mostly cannot be achieved in progressive pLGG by MEKi treatment alone [18, 21, 23, 31, 35]. CR-rates after MEKi treatment in the setting of progressive pLGG are by that equally low as in primary pLGG treated with conventional chemotherapy, where about only 1–2% of patients experience CR [36]. The low rates of CR indicate that parts of the tumor may not be targetable by the MEKi treatment alone, just as is the case with cytotoxic conventional chemotherapy. It is conceivable that this non-responding compartment, possibly quiescent through OIS and SASP [15, 37], is the source of the progression observed in some patients after EOT with targeted treatments. Supplementary strategies will therefore be needed to implement additional therapeutic regimens to improve treatment outcome and PFS of these patients. These could include e.g. the inhibition of other MAPK-related survival pathways like AKT/mTOR [38], or the use of senolytic drugs [37], both of which remain to be introduced into the clinical treatment of pLGGs.

PD was observed in 3/11 (27%) of the patients who stopped trametinib, within 4 months after EOT. The effect of rapid pLGG progression after MEKi withdrawal has already been described in the phase I clinical selumetinib study [17]: 10/39 (26%) patients experienced tumor regrowth within 6 months after EOT with selumetinib. Interestingly, seven of the ten patients progressing after EOT from the selumetinib study were not treated for the full planned 26 cycles (~ 25 months) due to toxicity or patient/physician preference possibly indicating that total treatment time (median of 12.5 months in the present cohort) may have an impact on PFS. Of note, re-initiation of trametinib treatment in one of the patients from the present cohort patients was able to stop progression and to induce a second disease stabilization (SD). This indicates that the tumor did not develop a bona fide resistance but retained its susceptibility towards the inhibitor, and that re-challenge with the same drug is an option, as has been reported for BRAFi [39]. The phenomenon of occasional rapid progression after MEKi treatment seems to be a class-specific effect, since it was also observed in the present study and is thus not restricted to selumetinib. This highlights the need for further preclinical work in order to define underlying mechanisms (e.g. modulation of oncogene-induced senescence, feedback modulations, etc.) and the best treatment modalities (treatment time, combination treatment, etc.) to overcome tumor growth rebound.

The mean dose of trametinib in our cohort was 0.03 mg/kg*day (± 0.009 mg/kg*day), which is in line with the recently published recommended phase II dose for pediatric patients six years of age and older [35]. Therapy related side effects occurred in 89% of our patients. This is comparable to results from a trametinib phase I clinical trial conducted in adult patients with advanced solid tumors, in which more than 90% of the trametinib treated patients developed toxicities [40]. The most frequent adverse events seen in our cohort were paronychia and different types of skin rashes, which were previously reports after MEKi treatments [17, 18, 35]. Dose limiting toxicities were documented in 32–40% of patients in the phase II selumetinib trial in pLGG and 3–12% had to discontinue treatment due to toxic effects [18]. Comparable to these findings, dose limiting adverse events were seen in 6/18 patients (33%), prompting repetitive treatment interruptions, and termination of treatment in 2/18 patients (11%). Similar rates of trametinib discontinuation due to toxicity have been reported in pediatric patients by Paul et al. [24]. We therefore conclude that some individual patients may display an unacceptable toxicity profile, in particular skin toxicity, underscoring the need for early and thorough dermatologist support in trametenib-treated children. We could not detect a correlation between dose reduction and response to therapy. This was also not reported in other case series on trametinib treatment of pLGG [21, 22, 24]. However, a correlation between dose reduction and change in response to therapy needs to be investigated by prospective trials.

In summary, we can confirm that oral trametinib treatment results in clinically meaningful responses in progressive pLGG patients with either *KIAA1549:BRAF*-fusion or *NF1* mutation. This strongly supports the evaluation of upfront MEKi treatment in the context of newly diagnosed patients with pLGGs within upcoming phase III clinical trials (e.g. the LOGGIC Europe trial).

Based on our data we conclude: (1) trametinib alone is probably not sufficient to induce tumor regression in all progressing patients, and drug combinations/additional targets need to be explored. (2) The underlying biological mechanisms behind the rapid progression of tumors in a fraction of patients after MEKi treatment need to be understood and addressed. (3) Most common adverse events associated with trametinib treatment are related to skin toxicity, requiring dose reduction or treatment discontinuation in individual cases, and warrant comprehensive skin care as well as early dermatologist support in affected children. These aspects highlight the need for alternative clinical strategies.

## Data Availability

The datasets generated during and/or analyzed during the current study are available from the corresponding author on reasonable request.
